# Supplementary material for: Human Papillomavirus Oncoproteins Confer Sensitivity to Cisplatin by Interfering with Epidermal Growth Factor Receptor Nuclear Trafficking Related to More Favorable Clinical Survival Outcomes in Non-Small Cell Lung Cancer
Source: Cancers (Basel). 2022 Oct 29;14(21):5333. doi: 10.3390/cancers14215333 (PMC9657246; doi:10.3390/cancers14215333)
Supplement: Supplementary file 1 [file cancers-14-05333-s001.zip › cancers-1941956-supplementary.pdf]

Supplementary Figure S1 Evidence for epidermal growth factor receptor (EGFR) nuclear translocation in NCI-H292 cells after Western blotting. After 100 ng/mL of EGF stimulus for 15 min and cell fractionation, SDS-PAGE and Western blotting were performed. EGFR with sc-03, Santa Cruz, was used for the detection of EGFR protein. Tubulin (α-Tubulin Antibody #2144) and histone (Histone H3 Antibody #9715) were used as nonnuclear and nuclear controls, respectively.

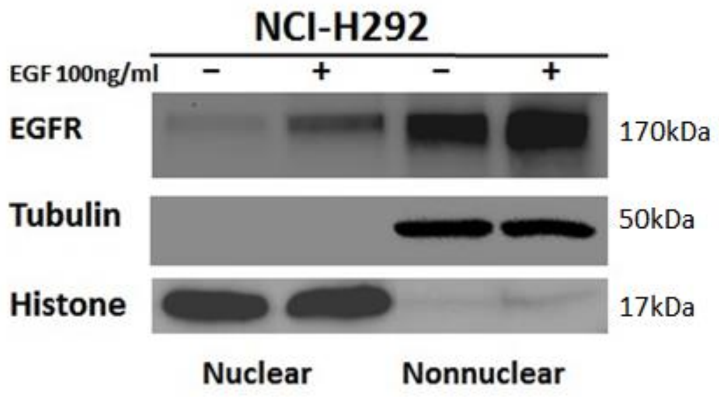

Supplementary Figure S2 Full pictures of the Western blots.

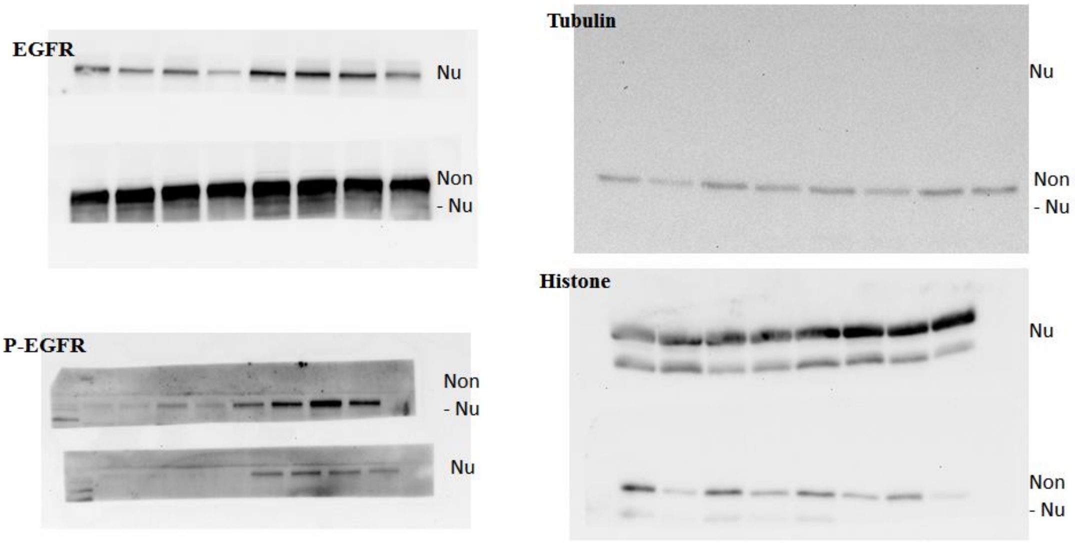

Supplementary Table S1 The detail methods for immunohistochemistry

**Steps**	**Methods**
Deparaffinization	1. Xylene 5mins x 3 times<br>2. 100% alcohol 3mins × 2 times<br>3. 90% alcohol 3mins<br>4. 80% alcohol 3mins<br>5. 70% alcohol 3mins<br>6. 50% alcohol 3mins
Antigen Retrieval	**1. For HPV 16/18E6 protein**<br>Heat-induced epitope retrieval in water baths with boiling citrate buffer for 20 min.<br>**2. For nEGFR protein**<br>Double-antigen retrieval method.<br>Heat-induced epitope retrieval (boiling citrate buffer for 5 min) plus enzyme digestion with 0.025% trypsin<br>(T4799, Sigma Aldrich) for 8 min at room temperature (RT).<br>**3. For mEGFR protein**<br>Enzyme digestion method with<br>pepsin (Digest-All™ 1, Thermo Fisher Scientific) for 10 min at 37 °C
Add primary antibody in slides	1. Incubation at 4 °C overnight using **anti-HPV16 E6/HPV18 E6** antibody (clone C1P5; Abcam), and NCL-EGFR-384 antibody (**nEGFR,** clone EGFR.25, Novocastra,Newcastle, upon Tyne, UK)<br>2. Incubation at RT using mouse anti-EGFr antibody (**mEGFR**, clone 31G7, Invitrogen, Breda, the Netherlands)
Add secondary antibody in slides	Second antibody (Dako K5007 HRP) 30 mins, RT
Immunoreactions	Using 3.3′-diamino-benzidine-tetrahydrochloryte for 10 min
Counterstain	Hematoxylin for 1min 20 second

Supplementary Table S2 Comparison of treatment response in 173 lung adenocarcinoma patients after tyrosine kinase inhibitors

Parameters	No	Median (m)	HR (95% CI)	*p*-value
**Total**				
**E6^{+}/tEGFR^{+}**	46	25.0	0.76 (0.45~1.27)	0.293
**E6^{-}tEGFR^{-}**	25	15.6	1	
**Older patients**				
**E6^{+}/tEGFR^{+}**	23	23.7	0.63 (0.31~1.29)	0.206
**E6^{-}tEGFR^{-}**	14	15.1	1	
**No brain metastasis**				
**E6^{+}/tEGFR^{+}**	23	31.7	0.72 (0.35~1.47)	0.364
**E6^{-}tEGFR^{-}**	15	19.3	1	
**Smokers**				
**E6^{+}/tEGFR^{+}**	12	52.1	0.37 (0.12~1.14)	0.083
**E6^{-}tEGFR^{-}**	6	15.9	1	
**Wildtype EGFR**				
**E6^{+}/tEGFR^{+}**	8	9.6	0.74 (0.26~2.08)	0.574
**E6^{-}tEGFR^{-}**	8	10.	1	

Cox proportional model

E6^{+}/tEGFR^{+} as **E6^{+}tEGFR^{+}**, **E6^{+}tEGFR^{-}** or **E6^{-}tEGFR^{+}**

Supplementary Table S3 Comparison of treatment response in 173 lung adenocarcinoma patients after radiation

**Parameters**	**No**	**Median (m)**	**HR (95% CI)**	***p*-value**
**Total**				
**E6^{+}/tEGFR^{+}**	41	23.7	0.71 (0.9~1.29)	0.256
**E6^{-}tEGFR^{-}**	17	10.4	1	
**Older patients**				
**E6^{+}/tEGFR^{+}**	22	21.6	0.58 (0.25~1.35)	0.204
**E6^{-}tEGFR^{-}**	8	9.3	1	
**No brain metastasis**				
**E6^{+}/tEGFR^{+}**	16	34.7	0.43 (0.17~1.12)	0.084
**E6^{-}tEGFR^{-}**	10	9.6	1	
**Smokers**				
**E6^{+}/tEGFR^{+}**	15	16.8	0.73 (0.29~1.89)	0.519
**E6^{-}tEGFR^{-}**	7	12.9	1	
**Wildtype EGFR**				
**E6^{+}/tEGFR^{+}**	18	4.32	1.02 (0.47~2.19)	0.961
**E6^{-}tEGFR^{-}**	11	7.63	1	

Cox proportional model

E6^{+}/tEGFR^{+} as **E6^{+}tEGFR^{+}**, **E6^{+}tEGFR^{-}** or **E6^{-}tEGFR^{+}**
